# Enhanced Field Emission of Single-Wall Carbon Nanotube Cathode Prepared by Screen Printing with a Silver Paste Buffer Layer

**DOI:** 10.3390/nano12010165

**Published:** 2022-01-04

**Authors:** Ruirui Jiang, Jianlong Liu, Kaiqiang Yang, Jing Zhao, Baoqing Zeng

**Affiliations:** National Key Laboratory of Science and Technology on Vacuum Electronics, School of Electronic Science and Engineering, University of Electronic Science and Technology of China, Chengdu 610051, China; 201711040120@std.uestc.edu.cn (R.J.); 201811022515@std.uestc.edu.cn (K.Y.); 202011022910@std.uestc.edu.cn (J.Z.)

**Keywords:** field emission, single-wall carbon nanotubes cold cathode, screen printing, silver paste buffer layer

## Abstract

A high emission current with relatively low operating voltage is critical for field emission cathodes in vacuum electronic devices (VEDs). This paper studied the field emission performance of single-wall carbon nanotube (SWCNT) cold cathodes prepared by screen printing with a silver paste buffer layer. The buffer layer can both enforce the adhesion between the SWCNTs and substrate, and decrease their contact resistance, so as to increase emission current. Compared with paste mixing CNTs and screen printed cathodes, the buffer layer can avoid excessive wrapping of CNTs in the silver slurry and increase effective emission area to reduce the operating voltage. The experimental results show that the turn-on field of the screen-printed SWCNT cathodes is 0.9 V/μm, which is lower than that of electrophoretic SWCNT cathodes at 2.0 V/μm. Meanwhile, the maximum emission current of the screen-printed SWCNT cathodes reaches 5.55 mA at DC mode and reaches 10.4 mA at pulse mode, which is an order magnitude higher than that of electrophoretic SWCNTs emitters. This study also shows the application insight of small or medium-power VEDs.

## 1. Introduction

Field emission cathodes are an ideal type of electron source for vacuum electronic devices (VEDs), which have the characteristics of radiation resistance, fast start-up, room temperature working, small size, and low cost [1]. Miniaturization and instantaneous start-up are development tendencies for VEDs. VEDs operating at millimeter and submillimeter wavelengths are of great interest for both scientific and commercial applications because of their stability to high temperatures and radiation [2,3,4]. However, the low stability at a high emission current is a key technical problem for field emission cathodes to be used in VEDs [5]. The Spindt cathode [4] and the thin-film field emission cathode [6,7] are the main research topics to make a high emission current. As early as 2000, Spindt et al. successfully developed a C-band traveling wave tube (TWT) with a Spindt-type field emission array cathode with a maximum emission current of 91.4 mA and the corresponding current density was 11.5 A/cm^2^ [4]. Then, they increased the emission current to 121 mA, and the maximum emission current density reached 15.4 A/cm^2^ [8]. The Spindt cathode has a high emission current, but the preparation cost is high [7], and it also has arc damage problems during operation [9]. Many difficulties need to be overcome for the Spindt cathode to be used in practice. The thin-film field emission cathodes, such as carbon nanotube (CNT) field emission cathodes, have lower production costs.

Since the CNTs were discovered in 1991 [10], their properties have attracted widespread attention from many scientists around the world. CNTs have excellent electrical and mechanical properties and can be applied in many fields. In particular, they have a high aspect ratio, good conductivity, and nano-level tips, which enable them to emit electrons for a long time at relatively low voltage. CNT cathodes can achieve high emission current density (~10^8^ A/cm^2^) [11]. The previous report indicated that the average current density of CNT cathodes reached 1.5 A/cm^2^, and the peak current density was as high as 12 A/cm^2^ [12], which could meet the current density requirements of many VEDs. However, their operation voltage and emission field were up to 10 V/μm, which was also easy to burn the gate mesh when they introduce to make electron gun. Reports also indicated that the CNT cold cathodes with turn-on fields of 2.5 V/um and threshold fields of 3.8 V/um had been obtained [13]. Liu et al. prepared graphene-CNTs hybrid by using one-step plasma-enhanced chemical vapor deposition (PECVD) without a catalyst. The field emission was stable with only an 8% decline after 10 h of continuous emission [14]. Studies have also reported the application of CNT cathodes to TWTs. A C-band TWT for satellite systems was reported but only with a small positive gain of 2.8 dB. That is because the CNT cathode on the TWT only delivered a total current of 4 mA in the pulsed mode, and the maximum current got in the collector was only 2.1 mA [15]. Li et al. made a CNT cathode-based electron gun assembled in a TWT. However, there was no power output in the TWT test due to the field emission current of the CNT cathode being too small to produce a power output [16]. Yuan et al. designed a conical cylindrical CNT cold cathode for a terahertz gyrotron with the maximum electron beam current of 28.2 mA in pulse state [17]. However, the operation voltage was as high as 12 kV.

Previous reports also showed that CNT cathodes were capable of having a high emission current [6]. To make a high field emission current with a relatively low emission field, SWCNTs were introduced in this work because they have highly crystalline structure with very few defective sites in their carbon network and are expected to lower the turn-on field (<1 V/μm) and stably emit electrons at a low driving voltage. The field emission current density of the SWCNTs could be higher than multi-wall CNTs, because of their thinner diameter, shaper tip structure, and fewer defects [18,19].

The cold cathode prepared by screen printing has the advantages of low cost, large preparation area, and is suitable for large-scale fabrication [20]. It firmly binds CNTs with the substrate [21], which can effectively reduce the contact resistance and increase the maximum emission current. The general screen printing method is to fully mix CNTs with organic slurry and then print on the clean substrate and form the cathode for one step. Paste mixing of CNTs in the printed cathode layer showed very poor electron emission characteristics because of their insufficient outcrop to the surface, random distribution, and possible organic residues. In order to remove these residues, tape pasting [22], mechanical friction [23], and laser irradiation [24] are generally used. Tape sticking and mechanical friction methods mainly remove the organic matter through the action of mechanical force. The tape adhesive method could both remove the residues on the surface of CNTs film and pull part of CNTs perpendicular to the substrate through the adhesive force. The mechanical friction method is to crush the residues on the surface through the action of mechanical friction so that more CNTs extend out of the film surface. The field emission properties of CNT films cathodes treated by these methods can be improved. However, it may also cause damage to the CNT film due to uneven mechanical force. It will also affect the emission uniformity of the whole cathode and reduce the adhesion between CNTs and the cathode substrate. When increasing the voltage applied to the anode, CNTs may fly out to the anode, resulting in short-time discharge and causing the device to be damaged. In this paper, silver paste was used as a buffer layer to enhance the adhesion and reduce the wrapping of organic matter on CNTs without undertaking any further surface treatment. After two-step printing, the CNT cathode is formed, and then high-temperature annealing is carried out to remove the residual organic matter.

## 2. Materials and Methods

Commercial single-wall carbon nanotubes (SWCNTs) (purchased from XFNANO, Inc., Nanjing, China, purity of SWCNTs: >95%, outer diameter: 1–2 nm, length: 5–30 mm) were mixed with ethanol and kept ultra-sounding for 2 h. Then it was baked at 60 °C until the mixture turned into sludge and fit for printing. The SWCNT sludge was screen printed on cleaned graphite substrates with a silver paste buffer layer. For comparison, the SWCNT cathode was also prepared by electrophoresis [25].

The substrate for preparing the SWCNT cathode is graphite. The impurities and dust on the substrate must be removed to prevent them from increasing the connection resistance between the SWCNT and graphite sheet. Firstly, we ultrasonically cleaned the graphite sheet with acetone for 10 min, and then change the cleaning solution to ethyl alcohol. Finally, we rinsed the substrate with deionized water and dry it in a constant temperature drying oven at 100 °C for 30 min. After drying, we get the needed cleaned and dried substrates.

**Figure 1:**
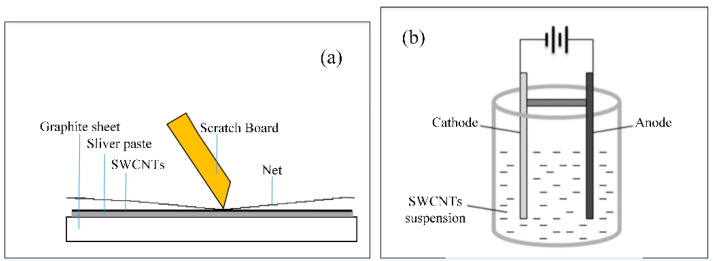
The schematic diagram of the preparation of SWCNT cold cathodes by (**a**) screen printing, and (**b**) electrophoretic deposition.

Before screen printing the SWCNT film, the first step is to screen print a layer of silver paste on the cleaned graphite substrate as a buffer layer, as shown in Figure 1a. Then, the graphite/silver paste is dried at 150 °C for 10 min to enhance the bonding force between them. Next, a layer of SWCNT sludge can be screen printed on the silver paste buffer layer in the same way as in the first step, and then dried at 200 °C for 20 min and follow annealed at 500 °C for 30 min under the protection of Argon to remove the organic impurities and increase adhesion to the substrate. When the temperature reduces to room temperature, take out of the sample and the SWCNT field emission cathodes are obtained. As for the electrophoretic SWCNT cathodes, the SWCNTs with the same weight ratio of Mg (NO_3_) · 6H_2_O were put into the isopropyl alcohol and ultrasonicated to form the SWCNTs suspension. Then the SWCNTs absorbed with Mg^2+^ were deposited on the cathode after applying voltage, as shown in Figure 1b. The distance of the cathode and anode was separated by a piece of alumina ceramic and kept at 10 mm. A constant potential of 100 V dc was applied for 40 min. This resulted in the deposition of the SWCNTs on the cathode electrode.

The characterization of field emission properties was measured with a diode structure (as the size of the SWCNT cathode is larger than the anode, we used the area of the anode as the effective emission area, anode-cathode gap: 300 mm, the diameter of the anode is 2 mm) that was placed in a vacuum chamber with the base pressure of about 1 × 10^−4^ Pa. An electric field was applied using the pulse (frequency: 10 Hz, pulse width: 1 ms) drive or the DC drive. Considering the turn-on field and threshold field corresponding to emission current density with 10 μA/cm^2^ and 1 mA/cm^2^ respectively. The structural properties of the screen-printed and electrophoretic SWCNT film cathodes were characterized by scanning electron microscopy (SEM) (FEI company, Hillsboro, OR, USA). Additionally, the morphology and microstructure of the SWCNTs used in the experiments were examined by high-resolution transmission electron microscopic (TEM) (FEI company, Hillsboro, OR, USA).

## 3. Results and Discussion

### 3.1. Structural Characterization

**Figure 2:**
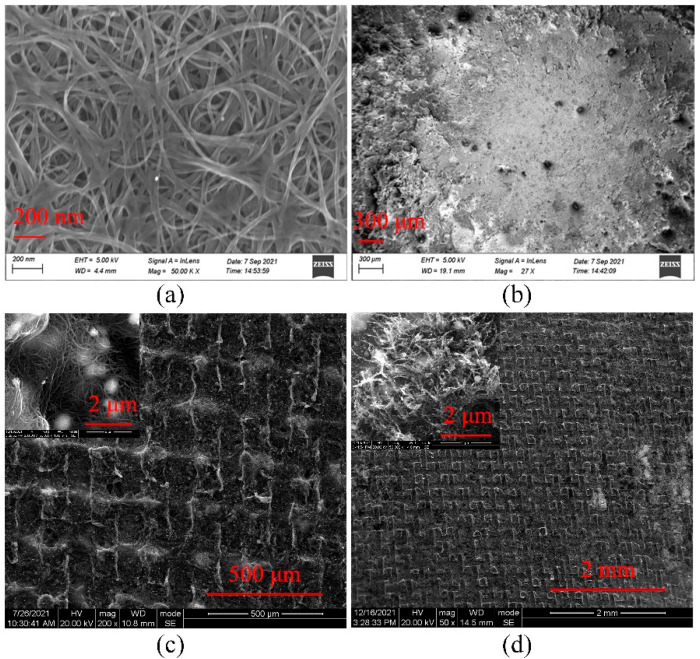
The SEM image of (**a**) the electrophoretic SWCNT cathode before and (**b**) after field emission test, (**c**) the screen printed SWCNT cathode before and (**d**) after field emission test. The insets of (**c**,**d**) are corresponding high-resolution SEM images before and after the field emission test.

The surface morphology of the screen printed and electrophoretic SWCNT cathodes before and after field emission test were analyzed by SEM. Figure 2a shows the morphology of electrophoretic deposited SWCNTs before the field emission test. It can be seen that the SWCNTs are uniformly covered on the surface of the substrate. After the field emission test with a large emission current, the electrophoretic SWCNTs are easy to separate from the substrate with a high applied electric field, as shown in Figure 2b. These separated regions will lose emission ability and the emission ability of the SWCNT cathodes would also be seriously reduced. Figure 2c shows the screen-printed SWCNT cathodes before the field emission test. It can be seen that the surface of the cathode is uniform. Besides, there are very few impurity particles distributed on the cathode surface, as shown in the high-resolution SEM image of the inset of Figure 2c, compared with the cathode prepared by traditional screen printing. Because of the silver paste buffer layer, the maximum emission current of the screen-printed cathode can be higher than the electrophoretic one. Figure 2d is the SEM image of the screen-printed SWCNT cathode after the field emission test. There is almost no change in the cathode surface morphology, from which we can see that the screen-printed SWCNTs have good contact with the substrate. There are numerous aligned SWCNTs are observed on the surface of the cathode. That is because of the electric field force between cathode and anode during the field emission process, which makes the SWCNTs lying horizontally on the substrate stand up one after another and become effective emission points, as shown in the high-resolution SEM image of the inset of Figure 2d. With the strong binding force provided by the silver paste buffer layer, the screen-printed SWCNTs will not be separated from the substrate under the applied external electric field, and remain to emit electrons under a high electric field. Compared with the electrophoretic deposited SWCNT cathode, the SWCNT cathode prepared by screen printing has better field emission performance.

**Figure 3:**
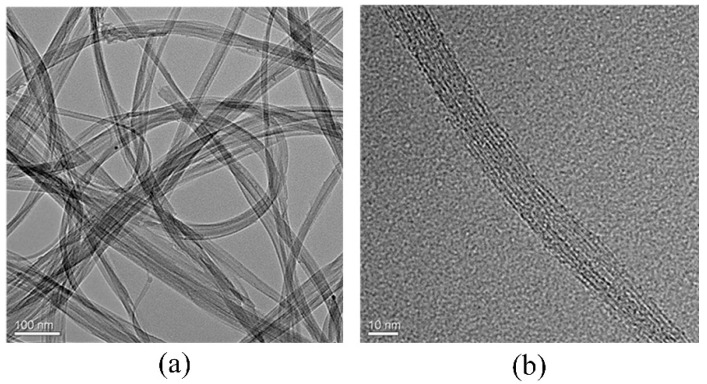
(**a**) The low-resolution TEM image and (**b**) the high-resolution TEM image of the SWCNTs.

Figure 3 is the TEM image of the SWCNTs. From Figure 3a we can see that the SWCNT sample consisted of long bundles of SWCNTs. However, due to the tendency of self-agglomeration of the SWCNTs, it is very difficult to separate a single SWCNT [19,26]. In addition, the emitters have pure SWCNTs without impurity particles on the surface of the nanotube bundles. The average diameter of the nanotubes is about 1–2 nm, as shown in the TEM image in Figure 3b.

### 3.2. Field Emission Characterization

The phenomenon of electron emission caused by reducing the barrier of cathode surface by using an external strong electric field is field-induced electron emission. The Fowler-Nordheim (*F-N*) model is widely employed to analyze electron quantum tunneling in various materials under the applied electric field. In this study, the field emission characteristics of SWCNTs were analyzed with the Fowler-Nordhiem (*F-N*) theory described by [27]

(1) $$J=A\frac{{\left(\beta ,E\right)}^{2}}{\varphi }e\;{-}^{\frac{B\varphi^{3/2}}{\beta E}}$$

where *J* is the emission current density, *E* is the electric field of the cathode surface, *β* is the field enhancement factor, *A* and *B* are constants (*A* = 1.54 × 10^−6^ A eV V^−2^ and *B* = 6.83 × 10^3^ V eV^−3/2^ µm^−1^), and *ϕ* is the work function of the cathode (here, a value of 5 eV is used for SWCNTs [28]).

By taking the natural logarithm, (1) can be given as the following linear equation:

(2) $$\ln(J/E^{2})=\ln\left(\frac{A\beta^{2}}{\varphi }\right)-\frac{B\varphi^{3/2}}{\beta E}$$

The plot of ln (*J*/*E*^2^) versus 1/*E* was plotted from *F-N* equation (2), which is a straight line called the *F-N* plot. The straight line indicates that electrons are derived from the quantum tunneling process.

The field enhancement factor *β* can be calculated from the slope (*k*) of the *F-N* plot and the work function of the cathode:

(3) $$\beta =-\frac{B\varphi^{3/2}}{k}$$

where *k* is the slope of the corresponding *F-N* plot. *β* represents the enhancement of the electric field of the emitter tip at the cathode. The emission current is higher with a larger *β* under the same external electric field.

Later, Forbes developed the *F-N* equation [29,30,31], developed as a more precise formal physical expression for the intercept correction factor. He proposed that the intercept correction factor in the *F-N* formula is a function of the electric field and discussed how to extract reliable emission areas from *F-N* plots. Therefore, only by accurately calculating the emission area can we get a more precise emission current density, which is important for comparing the field emission results from different field emission cathodes.

**Figure 4:**
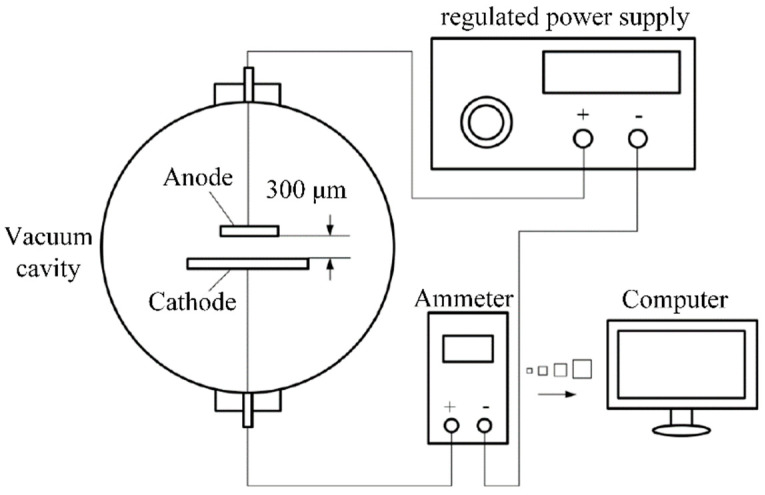
The schematic diagram of the electrodes configuration of field emission measurement.

The SWCNT cathode and a copper anode form a simple parallel plate diode structure, and the distance between cathode and anode is 300 μm. They are placed in a vacuum chamber with a base pressure as low as 1 × 10^−4^ Pa. An ammeter is connected in series in the circuit to measure the emission current, and the ammeter also is directly connected with the computer to record and save the emission current data. The schematic diagram of the test circuit is shown in Figure 4.

**Figure 5:**
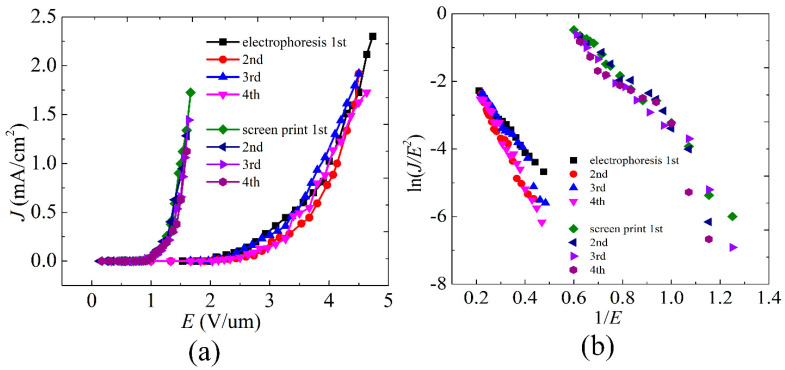
(**a**) The *J-E* curves of the screen-printed and electrophoretic SWCNT cathodes. (**b**) The corresponding *F-N* plots.

Figure 5a shows the *J-E* curves of the screen printed and the electrophoretic SWCNT cathodes. The turn-on field (*E_on_*) and threshold field (*E_th_*) of the screen-printed SWCNT cathodes are 0.9 V/μm and 1.5 V/μm, which is lower than many other reported screen-printed SWCNT cathodes [21,32]. This is due to the screen-printed silver paste buffer layer, which makes more emission points exposed to the cathode surface. The *E_on_* and *E_th_* of the electrophoretic cathode are 2.0 V/μm and 3.8 V/μm respectively. Compared with electrophoretic SWCNTs cathode, the screen-printed SWCNT cathode has a lower turn-on field. It can be seen in Figure 2c that there are many impurities wrapped on the electrophoretic SWCNTs, which should degenerate the field emission performance of the cathode. By fitting the slope of the *F-N* plot, we calculated the field enhancement factor of the screen printed SWCNT cathode is as high as 10,430. To obtain the reproducibility of I-V and F-E characteristics, each sample is measured repeatedly. Because the bonding force between the screen-printed SWCNTs and the substrate is stronger, the field emission curves of the screen-printed SWCNT cathodes are almost unchanged after repeated tests. Table 1 shows the results of the turn-on field and threshold field of screen-printed and electrophoretic SWCNT cathodes for each test.

**Figure 6:**
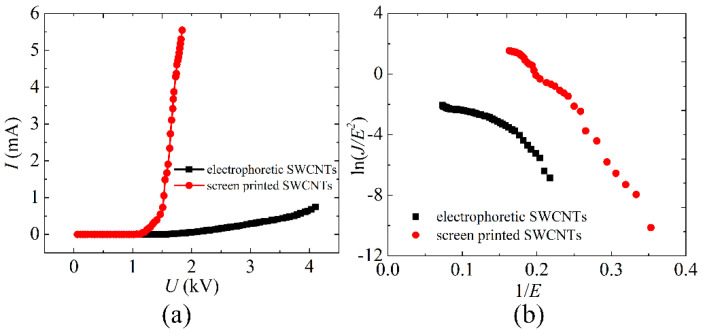
(**a**) The maximum emission current of the screen printed and electrophoretic SWCNT cathodes at DC mode. (**b**) The corresponding *F-N* plots.

The maximum emission current of the screen-printed SWCNT cathodes at direct current (DC) mode was 5.55 mA at 1.84 kV, corresponding to the emission current density of about 176 mA/cm^2^, which is much larger than that of the electrophoretic SWCNT cathodes, as shown in Figure 6a. Whereas the maximum emission current of electrophoretic SWCNT cathode was 0.75 mA at 4.1 kV, corresponding to the emission current density of about only 24 mA/cm^2^. Since the adhesion between the electrophoretic SWCNT film and the substrate is not as strong as that of screen printing, SWCNTs will separate from the substrate under the action of high voltage, as shown in Figure 2d. It is impossible to improve the emission current of electrophoretic SWCNT cathodes by continuing to increase the applied voltage. Figure 6b is the corresponding *F-N* plots of the screen-printed and electrophoretic SWCNT cathodes.

**Figure 7:**
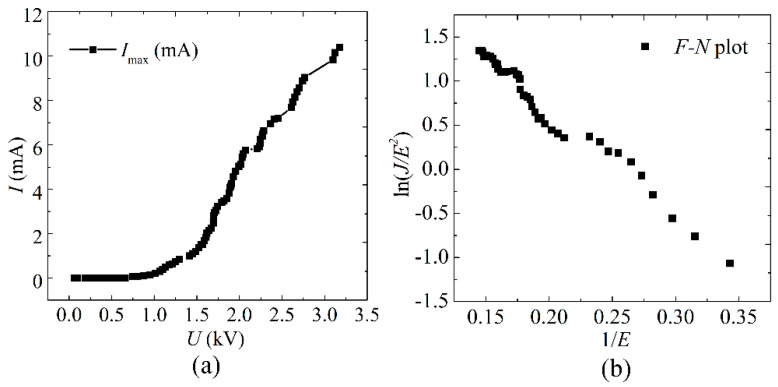
(**a**) The maximum emission current of the screen-printed SWCNT cathodes at pulse mode. (**b**) The corresponding *F-N* plot.

Figure 7 is the maximum emission current of the screen-printed SWCNT cathodes at pulse mode. The maximum emission current at pulse mode was about 10.4 mA at 3.23 kV, corresponding with the emission current density of 331 mA/cm^2^. In pulse mode, the maximum emission current is about two times higher than that of DC mode. Compared with the continuous bombardment of electrons on the anode at DC mode, the anode in the pulse state would produce less heat and have more heat dissipation time due to the alternative bombardment of emitted electrons. Therefore, a larger emission current can be obtained. With pulse driving mode, by adjusting the frequency and duty cycle, we can further increase the maximum emission current.

**Figure 8:**
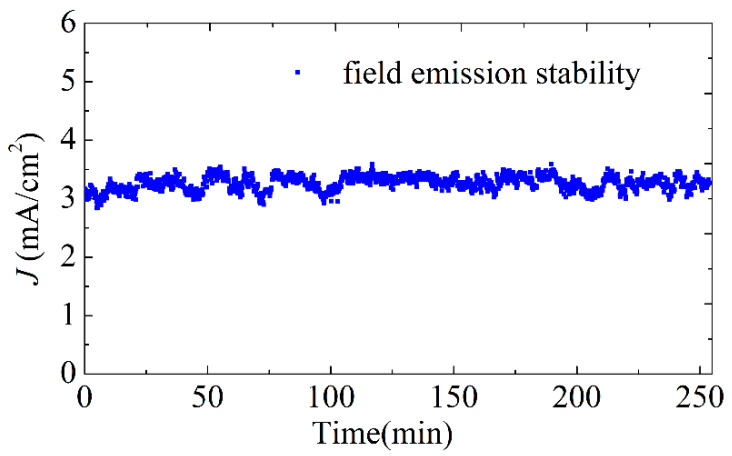
The field emission stability test (under the voltage of 810 V) of screen-printed SWCNT cathodes.

The DC field emission stability test of the screen-printed SWCNT cathodes is shown in Figure 8. The stability of the cathode is monitored at the current density of about 3.5 mA/cm^2^ under a voltage of 810 V. The emission current has relatively long time stability without obvious degradation, which reveals excellent field emission stability.

## 4. Conclusions

SWCNT cold cathodes with a silver paste buffer layer were prepared by screen printing. The silver paste buffer layer can not only significantly enhance the adhesion between SWCNTs and the substrate, but also increase the effective emission area on the cathode surface, so as to increase the emission current and reduce the operating voltage of the cathode. The experimental results show that the turn-on field of the SWCNT cathode is as low as 0.9 V/μm, and the field enhancement factor is 10,430. When the applied DC voltage is 1.84 kV, the maximum emission current reaches 5.55 mA, which is much larger than the SWCNT cold cathode prepared by electrophoresis. When the applied pulse voltage is 3.75 kV, the maximum emission current reaches 10 mA, which can meet the requirement of some small and medium-power VEDs. When the emission current is further increased, it can be widely used in many more VEDs.

## Figures and Tables

**Table 1:** Repetition tests of the screen printed and electrophoretic SWCNT cathodes.

Screen Printed SWCNTs Cathode	Turn-On Field/Threshold Field (V/μm)	Electrophoretic SWCNTs Cathode	Turn-On Field/Threshold Field (V/μm)
1st	0.9/1.5	1st	2.0/4.0
2nd	0.9/1.55	2nd	2.0/4.1
3rd	0.89/1.56	3rd	2.0/3.8
4th	0.95/1.57	4th	2.1/4.0

## Data Availability

The data presented in this study are available on request from the corresponding author.
